# An Assistive Technology Solution for User Activity Monitoring Exploiting Passive RFID

**DOI:** 10.3390/s20174954

**Published:** 2020-09-01

**Authors:** Bruno Ando, Salvatore Baglio, Salvatore Castorina, Ruben Crispino, Vincenzo Marletta

**Affiliations:** Department of Ingegneria Elettrica Elettronica ed Informatica, University of Catania, 95124 Catania, Italy; salvatore.baglio@unict.it (S.B.); salvatore.castorina@unict.it (S.C.); ruben.crispino@unict.it (R.C.); vincenzo.marletta@dieei.unict.it (V.M.)

**Keywords:** assistive technology, user habits monitoring, RFID, system characterization

## Abstract

Population ageing is having a direct influence on serious health issues, including hampered mobility and physical decline. Good habits in performing physical activities, in addition to eating and drinking, are essential to improve the life quality of the elderly population. Technological solutions, aiming at increasing awareness or providing reminders to eat/drink regularly, can have a significant impact in this scenario. These solutions enable the possibility to constantly monitor deviations from users’ normal behavior, thus allowing reminders to be provided to users/caregivers. In this context, this paper presents a radio-frequency identification (RFID) system to monitor user’s habits, such as the use of food, beverages, and/or drugs. The device was optimized to fulfill specifications imposed by the addressed application. The approach could be extended for the monitoring of home appliances, environment exploitation, and activity rate. Advantages of the approach compared to other solutions, e.g., based on cameras, are related to the low level of invasiveness and flexibility of the adopted technology. A major contribution of this paper is related to the wide investigation of system behavior, which is aimed to define the optimal working conditions of the system, with regards to the power budget, user (antenna)-tag reading range, and the optimal inter-tag distance. To investigate the performance of the system in tag detection, experiments were performed in a scenario replicating a home environment. To achieve this aim, specificity and sensitivity indexes were computed to provide an objective evaluation of the system performance. For the case considered, if proper conditions are meet, a specificity value of 0.9 and a sensitivity value of 1 were estimated.

## 1. Introduction

Society is currently undergoing gradual population ageing, which is increasing the incidence of health issues, including mobility, physical decline, and chronic diseases. As a result, the number of people needing support with daily activities is increasing [1]. Support can be provided by means of technological solutions monitoring the user activity rate, nutrition, and hydration quality [2]. As people age, they often experience reduced appetite and thirst [3], leading to poor habits that can have dangerous physical consequences [4,5,6,7,8]. These aspects are particularly important because proper activity (in terms of an active lifestyle) and correct nutrition/hydration can reduce the incidence of age-related traumas such as postural instability and falls, and more generally, physical decline. Conversely, a deficient lifestyle increases the chance of exacerbating mobility and physical issues, potentially resulting in greater dependency.

To introduce the current work, a review of the state-of-the-art research is provided, focusing on solutions aspiring at the increase of independence of people with some form of impairment. The analysis focuses on non-invasive solutions because these have been found to be the most favorable [9].

Both exercise and an active lifestyle have a relevant effect in improving the balance of older people, with a consequent reduction in the risk of falling [10]. Nevertheless, the elderly can often forget or neglect the physical activity they have been asked to perform. Typically, the responsibility to monitor the elderly is associated with caregivers, both professionals and family members. Recently, however, automatic solutions for the monitoring of daily activities, based on low-cost wearable devices, have been proposed. The primary aim of these solutions is to control fundamental parameters (especially geriatric parameters) such as the patient’s mobility, activity rates, and user’s habits and ADL (Activity of Daily Living) [11,12]. However, because falls may still occur, automatic systems can be used to promptly alert caregivers [12]. However, because falls may still occur, automatic systems can be used to promptly alert caregivers [13].

Another aspect in which technology has an important role deals with solutions aiming at increasing awareness or providing reminders to eat and drink regularly. Both apps and wearable systems have been proposed to monitor incorrect hydration or nutrition [14,15,16]. A first example, which has been developed into a commercial device, gathers information such as food and drink intake [17]. These solutions provide a mechanism to monitor a user’s abnormal behavior, which might indicate nutrition or dehydration scarcity and thus provide reminders to users and caregivers. Obli, which is essentially a balance for measuring fluid intake by weighing a bottle, is another example [18]. Obli uses visual and auditory signals to provide feedback when fluid intake is not adequate. The authors showed that the use of Obli increases the average fluid intake both 6 weeks and 6 months after the interventions began.

In general, it can be affirmed that in the field of assistive technologies (AT), non-invasive solutions are generally preferred [13,19,20,21]. Specific solutions that have attracted a significant amount of attention and shown increased applicability in the field of active assisted living exploit radio-frequency identification (RFID) technology. In the following discussion, examples of such applications involving the use of RFID solutions are given, ranging from assisted navigation and indoor navigation systems [21,22,23,24,25,26], to rehabilitation devices [27,28].

An example of navigation aid based on RFID technology is that reported in [21]. The system comprises an RFID reader and a sound system integrated on a traditional white cane, with the aim to help the visually impaired during the identification of surrounding objects. The RFID tags were integrated on tactile paving.

In [22], authors presented a prototype for an indoor location system using active RFID. Although RFID technology is not designed for accurate indoor location sensing, the proposed methodology, named the LANDMARC approach, shows that active RFID, using specific precautions, can be a valid candidate for accurate indoor location sensing.

An orientation system exploiting RFID for proximity detection to services was presented in [24]. The solution, aiming at improving the independent exploitation of unknown environments of people with visual disabilities, is presented with a metrological characterization aiming at finding the optimal conditions for system functionality.

Barman et al. [27] presented an interesting use of a sensor-enabled RFID system to monitor arm activity in daily life, focusing on the assessment of the sensor behavior when an object is handled.

In [29], S.S. Rao et al. used commonly available RFID technology for the accurate estimation of human activity tracking for applications such as retail, healthcare, work-place safety, and manufacturing.

RFID technology is clearly widely applied. Focusing on applications involving monitoring of user’s habits, devices presented in [14,18,27] are of interest. The solution in [17] allows monitoring of deviations from normal patterns of behavior which might indicate nutrition or dehydration scarcity, but does not monitor the intake of nutrients, handled objects, or explored areas. The solution in [18] provides an acoustic and visual alert in the case of inadequate fluid intake, but requires a static and fixed balance. Moreover, it does not provide the possibility to monitor users who sharing an environments but use different or private bottles. The solution in [27] uses RFID for monitoring arm activity in daily life. The system can read tags manipulated with one arm but does not consider objects manipulated with the other hand. This strongly reduces the usability of this system for the application addressed in the current work. The architecture proposed can be used in the case of single users in a home environment but does not provide a way to simultaneously monitor two or more users. Moreover, there is no association between handled object and users.

In the current paper, with regard to the aforementioned issues, an RFID based system designed to monitor user’s habits, such as the intake of food, beverages, and/or drugs, is presented and assessed. In addition, the same solution can be effectively applied to monitor home appliances, interaction with the indoor environment, and the activity rate.

To properly contextualize this activity, it must be considered that the presented device is a key component of the NATIFLife project [24]. This project aims at developing an innovative framework of assistive domotics which could improve the autonomy of elderly people. The development of an integrated platform of assistive technology, which is open to integration of traditional and innovative solutions, can produce an improvement in the life quality of the elderly and people with mobility impairments [30]. In detail, the project aims to assess user habits, activity rate, nutrition, and hydration, in addition to the use of home appliances.

The RFID system discussed in this work aims at integrating facilities to monitor food and beverage intake into the NATIFLife platform, combined with a unique identification of the user living in a specific environment. The main idea is to monitor the interaction between the user and generic targets (food, beverage, etc.) positioned in front of the user. To achieve this aim, the user wears an RFID reader, while each target of interest is labelled by a passive tag.

The advantages of the adopted technology compared other solutions, such as vision systems, switches, or other sensors, relate to its low level of invasiveness; good flexibility in terms of tag distribution, re-allocation, and quantity; ease of use and installation; and the unique identification of the user interacting with targets in the environment. All of these features are essential in effective assistive devices. It must be underlined that similar applications in the literature make use of static devices placed in the environment and invasive measurement systems installed in the target, and do not perform user identification (in the case of water intake, previous research has proposed the use of a sensorized bottle or the use of inertial sensors in the bottle itself). As previously stated, these solutions do not provide a flexible way to monitor a user’s behavior nor to understand which user is performing a given action.

A preliminary study was already presented in [24], which was mainly devoted to highlighting the procedure for the measurement of the power irradiated by the RFID system to properly define the optimal working area of the sensor itself. In particular, the subject investigated in [31] and further explored in this paper is the possibility to monitor/assist end-users during their interaction with objects that can be manipulated with the hands (food, beverage, drugs, furniture) using a low-cost device with a low level of invasiveness. This task is of primary importance for the monitoring of user habits/behavior in everyday life.

It should be noted that there is a lack of research focusing on quantifying the reliability of assistive technology (AT). Often, assessments are based only on a personal user’s evaluation provided by mean of questionnaires [32]. AT assessment is highly important, however. At a basic level, to fulfill the aim of AT to enhance the functional capabilities of users, an assistive device must be reliable, otherwise it is likely to be perceived as not compliant with user’s needs and the task to be accomplished.

In view of this need, the major contribution of this paper is hence related to the developed assessment procedure, which aims to define the optimal working conditions and characteristics of the assistive system, with particular regards to:

(i) The reader’s output power guaranteeing the requested antenna-tag reading range; in particular, the expected range is fixed to 30 cm with a target angular range of ±45°, which is a convenient working range related to objects managed by end-users.

(ii) The minimum inter-tag distance allowing close tag discrimination. It must be underlined that the latter investigation is valuable to assure a unique tag identification in the case of multi-tag occurrences, e.g., different targets positioned in the same working area in front of the user. Multiple tag detection, which typically represents the natural use of RFID-based devices, is a drawback in this scenario because it could compromise the correct discrimination between two close targets.

Moreover, to investigate the performance of the system in tag detection, particularly in terms of false positives, which may result in overconfidence in the solution, experiments were performed in a scenario replicating a homelike environment. To achieve this aim, specificity and sensitivity indexes were computed to provide an objective and reliable evaluation of the system performance. Typical values of these indexes, obtained during the experimental survey, are 96.5% and 92.1%, respectively.

Summarizing, new contributions of this work, both from methodological and experimental perspectives, with respect to [31], are as follows:

- The RFID system developed is described in detail to allow deep comprehension of its functionalities and peculiarities.
- A new experimental set-up and measurement procedure, adopted to estimate the optimal power conditions and guaranteeing the target range required by the addressed application, is reported. The new experimental set-up allows for deep characterization of the assistive system behavior for the specific case-study of an end-user interacting with objects manipulated by hand. Updated and extended results are also reported for the sake of completeness.
- A procedure aiming to assess the minimum inter-tag distance, thus reducing the mis-identification issue in the case of multiple objects positioned on the same line of work, is presented, with experimental results.
- Results obtained from tests conducted by end-users using the device in a homelike environment are presented. These preliminary results demonstrate the system reliability when used in a real scenario.

## 2. The RFID Based Measurement System

Although the measurement system was previously presented in [31], in this section a brief description of the device is reported for convenience.

**Figure 1:**
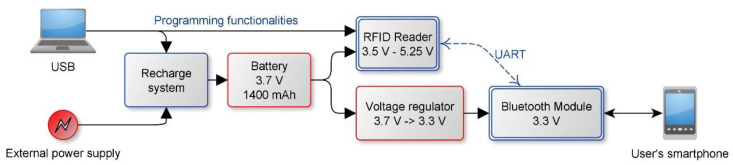
Block diagram of the hardware architecture.

The realized device is made up of a commercial RFID reader (M6E-MICRO by Thing Magic), a power management system, a 1400 mAh rechargeable battery (3.7 V) and a Serial to Bluetooth converter (RN42XVP-I/RM 2.1) to enable communication between the device and a smartphone. A schematization of the system is given in Figure 1.

Each item and piece of furniture to be monitored is fitted with a commercial flexible passive tag. Different types of tags are available in the market depending on the working surface on which the tag must be attached.

In the case considered of an assistive device supporting frail people in homelike environments, the device architecture is constrained by the application. In particular, dimensions should be compatible with a comfortable and wearable device, tags must be non-invasive and cheap, and the power budget should allow a device to operate for a reasonable time. On the basis of these considerations, for the case presented through this paper, the following choices were made:

- antenna maximum dimension was fixed to 3 cm by 3 cm.
- the selected tags were: AD-227m5 (Avery Dennison).

**Figure 2:**
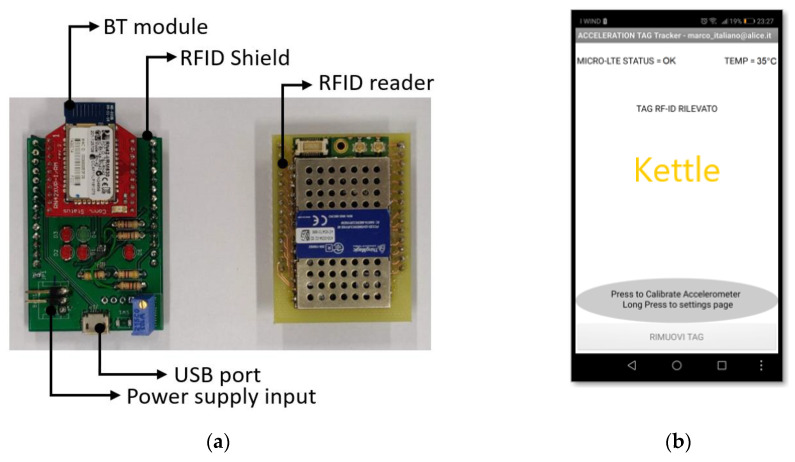
Device overview: (**a**) real view of the device; (**b**) the Android interface.

To control the system, an Android app (Figure 2b) was developed. It is comprises routines to guarantee continuous communication with the RFID reader and a basic interface allowing users to manage or associate unknown tags. In detail, it allows associating a unique nickname (which specifies the detected objects) with each tag and sending the tag EPC (Electronic Product Code), nickname, and detection time to an HTTP server. More details related to the system operation are available in [32].

**Figure 3:**
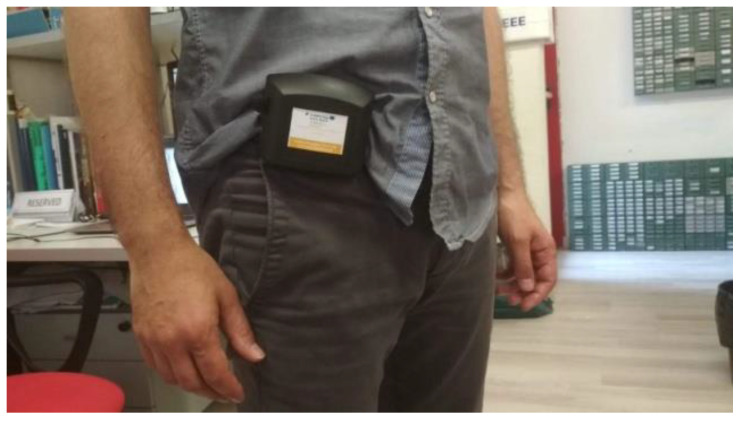
The system worn by a real user.

The device developed for this application is shown in Figure 2a, and the system worn by a user is shown in Figure 3.

## 3. The Experimental Set-Up and Results of the System Characterization

As previously introduced, the main goal of the RFID system is to individualize the end-user proximity to furniture and/or food and drink in an environment replicating a home. This assessment is necessary to assess the user’s activity rate, habits, hydration, and nutrition.

To correctly detect the target (furniture/tool/food/drink) the user is interacting with, two main aspects need to be addressed: the maximum operating range of the system in terms of the maximum allowable distance between the antenna worn by the user and the tag applied to the target; and the minimum distance between two tags (identifying two different targets) thus avoiding any cross-detection of multiple tags in a specific reading range. The idea of this system is to assess users’ habits and to monitor their actions, e.g., if a user is using a cooking tool or handling a bottle of water. Each object is labelled by a dedicated tag with a unique code so that user interaction with that object can be identified. A problem arises in that many of these objects can remain in the same area. If the RFID system detects two or more objects (multi-object detection) simultaneously, the system will not be able to understand which object the user is interacting with. This is the reason multi-object (cross) detection should be avoided.

It should be noted that objects are not required to be positioned in specific locations. The only constraint to be fulfilled is the minimum distance between two tags, which allows for reliable tag discrimination.

The above features are usually constrained and must be compliant with the needs associated with the practical use of the device. In particular, for the considered case, the target operating range of the device (antenna-tag distance) was fixed to 30 cm, which is the typical range at which an object is easily used by the hands, and the minimum inter-tag distance was fixed on the basis of the characterization results, as shown in the following sections.

The developed solution offers different degrees of freedom to fulfil the above specifications, such as the antenna dimensions, the tag type, and the reader output power. As previously discussed in Section 2, the architecture (antenna, tag) was chosen to be compliant with the practical needs of usability and acceptability. In the following section, measurement strategies adopted to fix the optimal reader’s output power, thus fulfilling the antenna-tag distance specification, are presented. Subsequently, results of investigations to determine the minimum inter-tag distance are discussed in Section 3.1.

### 3.1. Investigations on the System Reading Range vs. Reader’s Output Power

The aim of this measurement strategy, as previously noted, is to ensure the target reading range while minimizing the device’s power consumption. In particular, the RSSI (Received Signal Strength Indication) is used to identify the areas in which the irradiated power is sufficiently high to power the passive tag. Specifically, four different values of the reader’s output power were investigated, namely 25, 20, 15, and 10 dBm.

**Figure 4:**
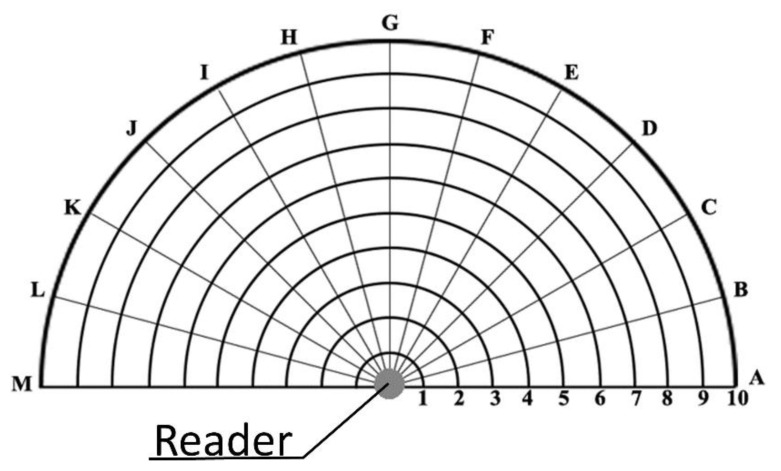
Measurement points used during the first assessment.

Measurements were taken along the grid shown in Figure 4. Each point in the chart, for a given angle, is spaced from the previous point by 5 cm. The addressed distances range from 5 to 50 cm. The addressed angles are in the range of 0–180° with a step of 15°. In total, 130 measurement points (intersections) were investigated. There are two main reasons why the measurements were taken along a semi-circumference rather than the total circumference: (1) the back radiation gain of the adopted antenna is negligible with respect to the front radiation gain; and (2) the final application does not expect readings for objects placed behind the user wearing the device.

The measurement protocol consisted of the following steps:

1. Fixing the reader’s output power, starting with the maximum value (25 dBm);
2. Placing the tag at a fixed distance from the antenna (e.g., 5 cm, combination 1-C);
3. Moving the tag to each of the 13 angular positions while maintaining the distance from the reader antenna (e.g., 1-A, 1-B, …, 1-M);
4. Measuring the tag emitted power for each measurement point;
5. Increasing the distance from the emitter by 5 cm and repeating points 3 and 4;
6. Repeating every step with a different reader’s output power, decreasing by 5 dBm until the requirements are not met.

During the duration of the experiment, both the RFID reader and the passive tag were permanently placed in wooden supports in such a way to increase the set-up reliability. In particular, the reader was placed in the position marked by the filled circle at the bottom of the diagram.

This procedure allows the construction of an irradiation diagram representing the output power as a function of the angle/distance parameters, guaranteeing the detection of the tag placed at each of the measurement points. This strategy led to the identification of the minimum power guaranteeing the desired reading range of ~30 cm with a detection angular range of ±45°.

This operating domain was defined by the specific application context, considering a typical user–target interaction area in the case of handled objects (food, beverage, drugs).

This preliminary phase, which was conducted in a laboratory set-up, was carried out taking into account all of the necessary steps to avoid interference caused by material placed between the reader and tag, or strong electromagnetic interference in the surrounding area.

The obtained results were arranged to show the tag irradiated power when it was placed at the measurement points shown in Figure 4, thus facilitating the comprehension on the combination of angle/distance allowing reliable tag detection. Figure 4 represents a schematization of the points at which measurements were taken. Each of these points is associated with a given RSSI.

**Figure 5:**
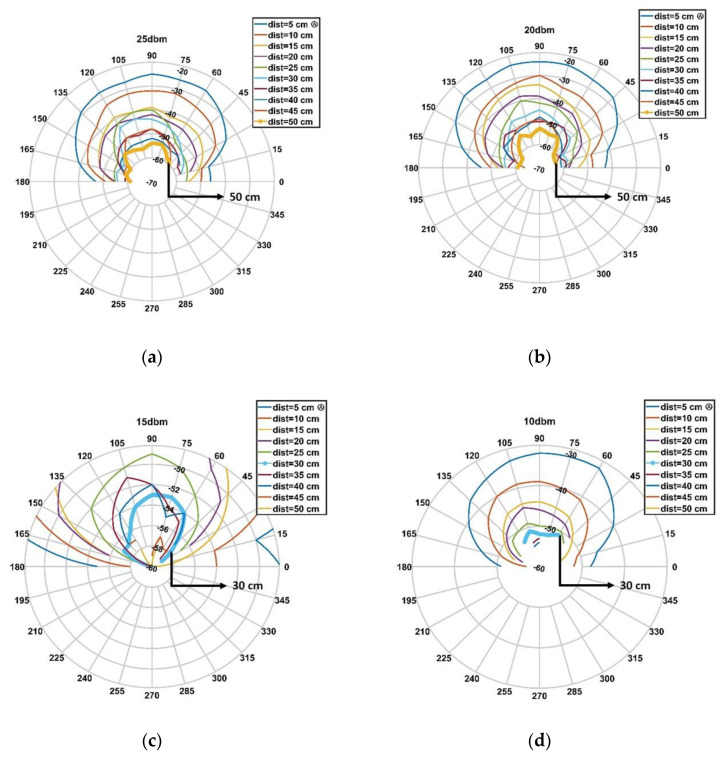
Characterization results. The tag irradiated power: (**a**) when the output power is fixed to 25 dBm, (**b**) when fixed to 20 dBm, (**c**) when fixed to 15 dBm, and (**d**) when fixed to 10 dBm.

Results obtained for each of the considered power levels are shown in Figure 5. This figure is built as an interpolation of the RSSI measures as a function of the reader power, reader-tag distance, and angle.

An analysis of the plots reported in Figure 5 allows the following conclusions to be drawn:

- 25 dBm output power (Figure 5a): this power value produces a reading range up to 50 cm in the whole cone of acceptance (±45°), as indicated by the bold line highlighted in the plot.
- 20 dBm output power (Figure 5b): this power setting shows similar behavior compared to the previous setting.
- 15 dBm output power (Figure 5c): analyzing the target range, which is highlighted by the bold line (30 cm), full coverage is guaranteed in the entire angular range of operation (±45°). Nevertheless, greater distances, namely 40, 45, and 50 cm can still be achieved. However, it must be clarified that these values are not guaranteed in the desired angular range but are spotted measures within the measurements’ points. These results mean that this power level (i.e., 15 dBm) is the optimal choice for the application’s requirements. For the sake of clarity, the plot reports only results related to a distance range of 25–50 cm.
- 10 dBm output power (Figure 5d): this power level was investigated to understand if a further power reduction may have been possible. As clearly shown by the bold line, the target reading range is not guaranteed within the angular range of operation.

In conclusion, it can be affirmed that the optimal reader’s output power, covering the desired interaction area, is equal to 15 dBm, which allows a suitable user–target interaction within the whole operation area (defined as a distance of 30 cm in the range −45° to 45°). Moreover, both from a theoretical analysis and testing, with the selected power of 15 dBm, the battery life was estimated to be around 5 h. The power required by the RFID module, in reading mode, is around 1 W/h. Using a battery of 1400 mAh with 3.7 V, the total available power is 5 W/h. It should be noted that any smart control integrated into the device that took into account prolonged inactivity (resting or sleeping) could drastically improve the battery life; however, this task is outside the scope of the current work.

The first conclusion can hence be summarized as follows: in the case in which the end-user handles objects in the above defined working range, the system assures the identification of user–target interactions.

The above working range was clearly defined in a conservative manner, ensuring a full range of tag identification. As can be observed by the results obtained, in the central area of the working range the system is able to detect tags positioned at a distance of up to 50 cm. This consideration affirms that, in a real scenario, where the interactions between end-users and items (beverage, food, etc., positioned on a working table) have to be monitored, targets should be placed at a minimum distance of 50 cm from the line of action of a potential end-user. The above assumption is confirmed by the case study presented in Section 4.

### 3.2. A Measurement Strategy to Assess System Behavior in Terms of Undesired Multiple Object Identification (MOI)

As previously described, an aspect worthy of consideration in the development of assistive devices is the maximization of user confidence and trust. Consequently, the reliability of the system is a mandatory characteristic to avoid users’ misplaced confidence. A specific characteristic that should be addressed by the system under investigation is high robustness against MOI. To guarantee the expected reliability during monitoring of user activities inside a living environment, the system should identify one tag at a time and MOI must be avoided.

In particular, the above investigation is essential in the case in which the RFID system is used to identify objects positioned next to the end-user, e.g., to notify the user of the presence of the target or to monitor the closeness of the user to specific targets (user traceability). In this case the user is not required to necessarily handle the target.

In this section an assessment procedure based on a dedicated measurement survey is proposed to analyze the system behavior in the presence of more than one tag within the working range of the system, allowing the minimum distance between tags, ensuring high selectivity of the identification procedure, to be determined. This is essential to properly set the position of tags associated with different services or tools in a real scenario.

**Figure 6:**
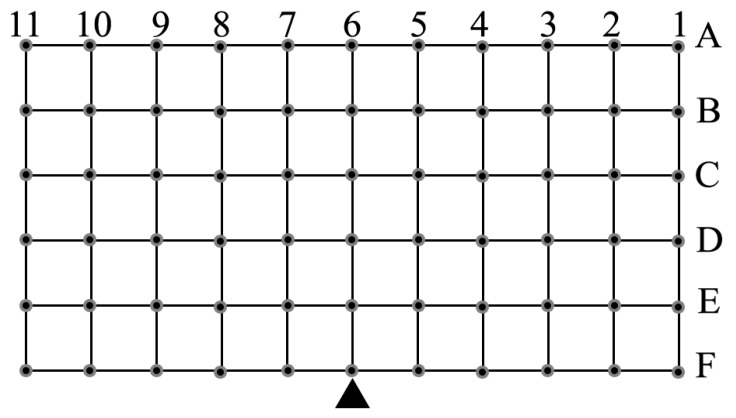
Working area adopted to assess the system behavior in terms of multiple object identification (MOI). Dimensions are 100 by 50 cm with each step measuring 10 cm.

The measurement survey was performed by a dedicated set-up represented by the working area shown in Figure 6.

The experiment consists of observing the system behavior in the case in which two tags are positioned within the rectangular area. The main task is estimation of the minimum inter-tag distance that ensures MOI is avoided.

Circles represent the measurement points spaced from another by 10 cm. The black triangle at the bottom defines the reader position.

For each measurement, one tag (labelled “#1”) is fixed in the position defined by (row (i), column (j)), chosen as the reference position, and a second tag (labelled “#2”) is moved along all of the other points belonging to the same row. For the sake of completeness, it must be noted that only combinations of tags belonging to the same row (lines of operation in front of the user) were investigated, which represent the worst case in terms of MOI.

**Figure 7:**
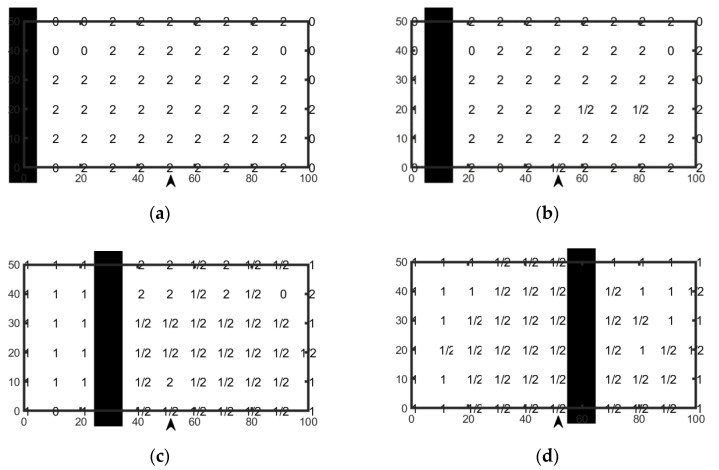
Results showing the tag detection area as a function of the position of the two tags. In particular, tag #1 is fixed on the black cell of each row, chosen as the reference position, while tag #2 is moved to all of the other positions of the same row. The position of the reader is marked by the arrow. In (**a**–**d**) cases for different positions of tag#1 are presented.

The obtained results in terms of detected or missed readings are shown in Figure 7. As an example, Figure 7a shows results related to the case in which measurement surveys have were accomplished by successively positioning fixed tag #1 in the first column of each row while tag #2 was moved on the corresponding row from column 2 to column 11. For each of the combinations produced by means of this strategy, the plot reports a “0” when the system did not detect the presence of tags, a “1” or a “2” when the system detected one of the two tags, and a “1/2” when both tags were detected (MOI). The dark vertical cells show the position of the fixed tag #1.

Since the aim of this measurement procedure was to identify the areas in which MOI can occur, our attention must be focused on spots labelled “1/2”, representing the conditions in which a cross-detection of multiple tags was detected. To avoid MOI, these combinations should be avoided.

As expected, in the case in which tag #1 is outside of the reader working range, only tag #2 is detected (see Figure 7a). In the case in which tag #1 is positioned close to the working area MOI may occur (see Figure 7b), and in the case in which tag #1 is positioned inside the working range, a large number of MOI incidents were identified.

As already evidenced in previous analysis, tags positioned within a cone smaller than ±45° can also be detected if the reader-to-tag distance is greater than 30 cm.

Although a small number of exceptions were highlighted by the performed investigation, the obtained results confirm the need to maintain the inter-tag distance above 60 cm to avoid MOIs.

Asymmetries in the device behavior can be assumed to be dependent on the asymmetric irradiation diagram shown in Figure 5. This result is due to intrinsic asymmetries of the device.

It must be noted that these results are not related to the need to detect objects handled by end-users. Rather, they relate to the opportunity to use the proposed technology to detect and recognize targets (services, landmarks, etc.) adjacent to end-users.

## 4. The Device Behavior in a Real Scenario

Experiments presented in this section aimed to assess the system reliability when it is used in a scenario simulating a home environment.

### 4.1. Monitoring User Handling of Tagged Objects

As previously mentioned, the task to be assessed by the proposed methodology is the monitoring of the interaction between objects and a user handling these objects. A typical scenario, investigated in the following discussion, is a user standing in front of a table (working area), taking and using objects positioned on the table (water, drugs, food, etc.). In this case, the line of sight between tags and the antenna is guaranteed by a convenient positioning of the reader antenna on the user’s body and by avoiding shielding layers between tags and the reader.

This assessment was conducted by fulfilling the requirements depicted in Section 3 in terms of object positions and the user handling range.

**Figure 8:**
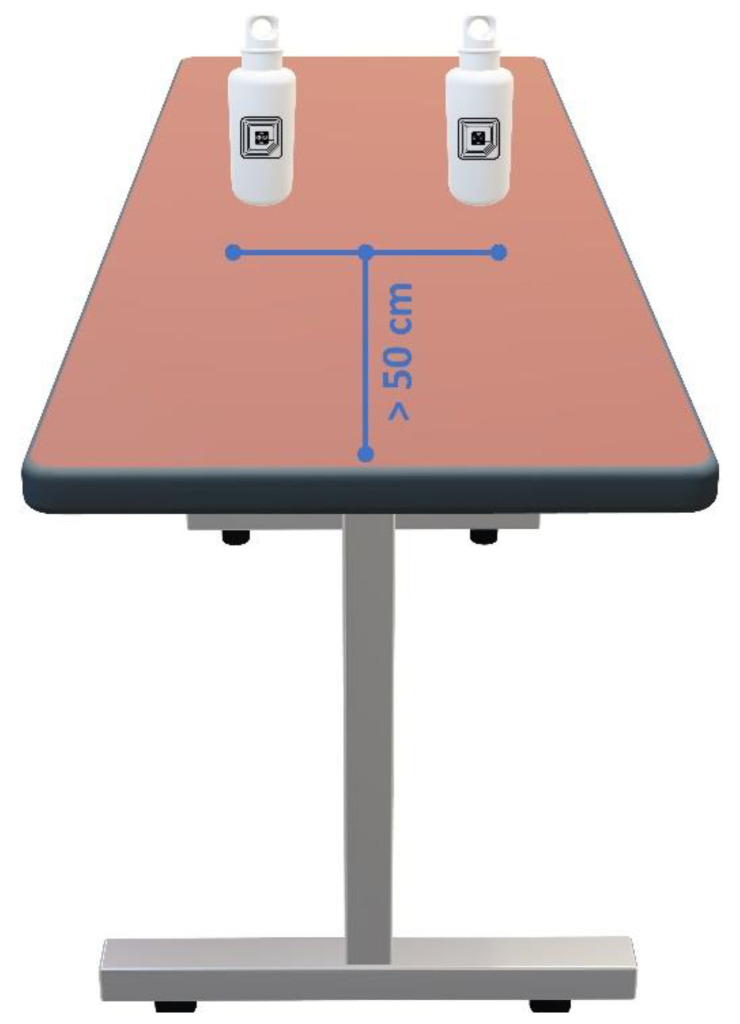
A schematization of the experimental setup which was used for the assessment of the device in the case of handled objects.

The homelike environment was simulated by considering a working surface (e.g., a table) on which two targets were positioned. The users were asked to stand still in front of the table, with objects placed at a distance of 50 cm from the user’s hip (the RFID reader was worn on the right side of the user’s hip and the objects were represented by two bottles of water), and to take and use the objects, one at a time, five times each. For each use, the system output was acquired and the following cases were addressed: the system detects the handled object (this case was considered as a true positive, TP); the system does not detect any object (a false negative, FN); and the system detects the wrong object (a false positive, FP). A schematization of the experimental setup is shown in Figure 8.

In particular, to ensure that the system only detects the tag during genuine user–target interactions and not while the user is approaching the table, tagged objects were positioned at a minimum distance from the user’s line of action of 50 cm. Considering that the reader was worn at the right side of the user’s hip, objects were positioned at a distance greater than 45 cm from the table’s edge. During the experiment, users emulated typical actions associated with that specific object; as an example, if the object under investigation was a bottle, the associated actions included opening the bottle and subsequent pouring of water. Clearly the device does not know if the user is actually drinking, but the approach is based on the idea that if the user takes the bottle it his intention is to do so.

For this first assessment, 10 users (aged between 22 and 39, with different heights and weights) were involved, all of whom repeated the experiments five times per object. Users were given brief instructions about how to mimic gestures typically performed by frail people, fulfilling the need of bringing the object within the system operating range (30 cm, ±45°). This phase is extremely important because gestures normally performed by healthy and young people may fall outside the reading area of the device.

During the experiments, each participant signed an informed consent regarding the purpose of the study and working conditions. Moreover, every precaution was taken to ensure user safety during experiments.

The object detection was monitored by means of the Android application introduced in Section 2.

**Figure 9:**
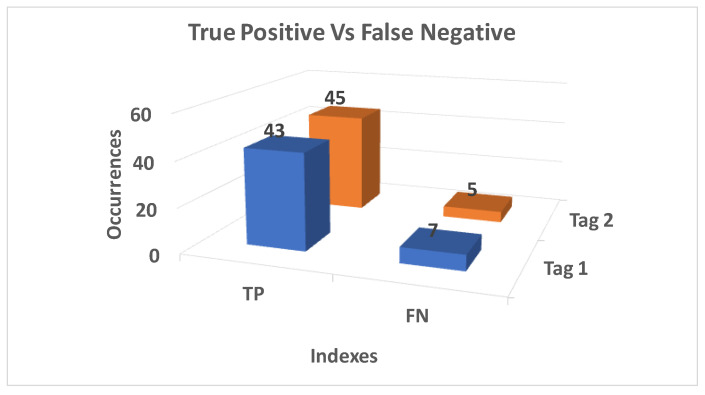
True positive (TP) and false negative (FN) behavior in the experiment aiming at monitoring user handling of tagged objects. The number on top of each column represents the total number of occurrences for that specific index.

Results for this experiment are shown in Figure 9.

First, as evidenced by Figure 9, no false positives (FP) were identified. This can be explained because the experiment was set up according to the rules defined in Section 3.1: placing the objects at a distance greater than 50 cm from the user ensures their detection only during their use. Moreover, the low number of FN demonstrates that tags were infrequently not detected during their use.

The above results confirm the reliability of the system in the case in which objects to be handled are positioned by fulfilling the constraints defined in Section 3.1.

### 4.2. Detecting Items Next to End-Users Avoiding MOI

The main goal of this experimental test is not related to the need to detect objects handled by end-users; rather it relates to the opportunity to use the proposed technology to detect and recognize targets (services, landmarks, etc.) adjacent to end-users. Hence, a second assessment was conducted by analyzing the capability of the system to detect the correct item when two or more targets were positioned in the user’s surrounding area.

To mimic a possible scenario in which the aforementioned situation can occur, the following two possible scenarios were considered:

- The user is exploring an area where appliances to be monitored are close to each other;
- User activity needs to be monitored by detecting his/her transition to a target placed throughout the environment.

Although different in appearance, both scenarios share the same problem: two or more tags can be possibly placed close to each other, increasing the probability of MOI.

**Figure 10:**
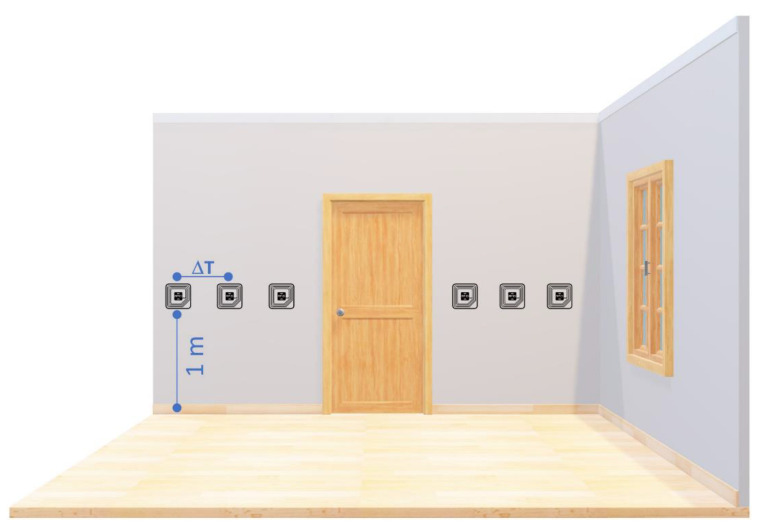
A schematization of the scenario adopted for the sake of the second assessment.

To address this issue, an experimental scenario was prepared (Figure 10). Tags were placed on a wall at a fixed height (1 m from the ground) and with changing inter-tag distance (∆T). The ∆T distances addressed were 60, 50, and 40 cm. The experiments were conducted as follows:

- the device was positioned at the users’ hip in such a way to direct the antenna toward the tag;
- users, always starting from the same tag, followed a path (highlighted on the room’s floor) maintaining a constant distance, 40 cm, from the wall (and hence from the tag) and a slow pace;
- for each transition through a target, an experimental supervisor took note of the following cases:
  ○ tag read: considered a true positive (TP);
  ○ tag not read: considered a false negative (FN); this can be generated by a reader-tag distance greater than the nominal distance, a reduced reading range caused by anomalous working conditions, etc.
  ○ tag correctly read and consecutive/previous tag also read: considered both a TP and a false positive (FP).

During the transition between two consecutive tags, an experimental supervisor took note of the following case:

- any tag read: considered a true negative (TN).

The users involved in these experiments were the same as those involved during the experiment described in Section 4.1. Each repeated the experiment five times for each ∆T combination.

Elaborating data achieved by a binary classification approach, the following indexes were used to assess the system performance:

Sensitivity:

(1) $$S_{e}=\frac{TP}{TP+FN}$$

Specificity:

(2) $$S_{p}=\frac{TN}{TN+FP}$$

Moreover, to also evaluate the incidence of the *MOI* in the system, the specificity index was normalized as follows:

(3) $$S_{p_{norm}}=\frac{TN}{TN+FP}\cdot \left(1-\frac{MOI}{TT}\right)$$

where *TT* stands for Total Trials.

**Figure 11:**
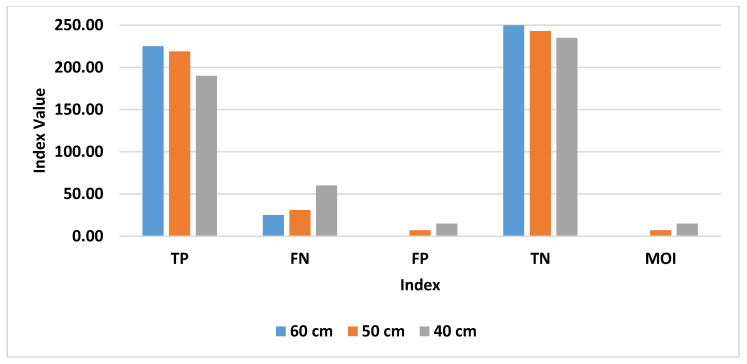
Raw indexes for each inter-tag distance addressed.

**Figure 12:**
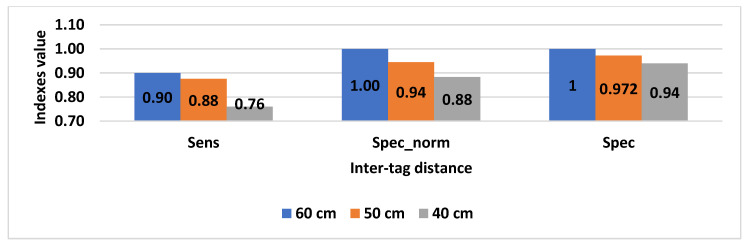
Sensitivity, specificity, and normalized specificity for each considered ∆T.

Results concerning the aforementioned indexes are shown in Figure 11 and Figure 12.

Figure 11 allows a comparison to be made with the results discussed in Section 4.1. In particular, as the inter-tag distance is reduced, there is a clear increment in the FN, FP, and MOI values, while the TP value decreases. This can be interpreted as a general deterioration in the system capability to clearly detect and recognize targets next to end-users.

This trend is further confirmed by results shown in Figure 12, where a general deterioration of the system performance, as the inter-tag distance is reduced, can be clearly noted.

It can be finally affirmed that the results of the assessment strategy confirm the results of Section 3, making it eligible to conduct an assessment of this system in the NATIFLife living lab and involving real elderly users.

## 5. Conclusions

Population ageing is leading to exponential growth of serious health issues, including hampered mobility and physical decline. Numerous solutions have been proposed to reduce the societal effect of such phenomena, such as gait training in the case of falls, or reminders in the case of nutrition and hydration. These aspects are particularly important because proper activity (in terms of an active lifestyle) and correct nutrition/hydration can reduce the incidence of age-related traumas such as falls, postural instability and, more generally, physical decline. In these scenarios, the use of technology can result in a better outcome of the proposed solutions because such approaches provide an objective estimation of user’s habits, activity rates, or the use of tools, food, and/or equipment.

As a possible advance in this domain, an RFID-based system aimed at monitoring user’s habits, such as the use of food, beverages and/or drugs, use of equipment, exploitation of the indoor environment, and activity rate, is presented.

Advantages of the proposed system relate to its low level of invasiveness, flexibility, and intrinsic user identification feature.

For the targeted scenario, specific attention was placed in the proposed approach on the optimization of the system characteristics in terms of physical dimension, power consumption, and reading range.

Moreover, to avoid incorrect identification resulting in misplaced confidence in the solution, an assessment procedure was conducted aiming at evaluating the system reliability when used in a scenario simulating a domestic area. Specificity and sensitivity indexes demonstrated the robustness and reliability of the solution. If proper conditions are met, a sensitivity index value of 0.9 and specificity index value of 1 can be achieved.

On the basis of these obtained preliminary results, future efforts will be made to test the system in a real environment, including the living lab established for the NATIFLife project.
